# Anabolic steroids for rehabilitation after hip fracture in older people

**DOI:** 10.1590/1516-3180.20161345T2

**Published:** 2016-09-26

**Authors:** Vaqas Farooqi, Maayken E. L. van den Berg, Ian D. Cameron, Maria Crotty

## Abstract

**BACKGROUND:**

Hip fracture occurs predominantly in older people, many of whom are frail and undernourished. After hip fracture surgery and rehabilitation, most patients experience a decline in mobility and function. Anabolic steroids, the synthetic derivatives of the male hormone testosterone, have been used in combination with exercise to improve muscle mass and strength in athletes. They may have similar effects in older people who are recovering from hip fracture.

**OBJECTIVES:**

To examine the effects (primarily in terms of functional outcome and adverse events) of anabolic steroids after surgical treatment of hip fracture in older people.

**METHODS:**

*Search methods:* We searched the Cochrane Bone, Joint and Muscle Trauma Group Specialized Register (10 September 2013), the Cochrane Central Register of Controlled Trials (CENTRAL) (The Cochrane Library, 2013 Issue 8), MEDLINE (1946 to August Week 4 2013), EMBASE (1974 to 2013 Week 36), trial registers, conference proceedings, and reference lists of relevant articles. The search was run in September 2013.

*Selection criteria:* Randomized controlled trials of anabolic steroids given after hip fracture surgery, in inpatient or outpatient settings, to improve physical functioning in older patients with hip fracture.

*Data collection and analysis:* Two review authors independently selected trials (based on predefined inclusion criteria), extracted data and assessed each study's risk of bias. A third review author moderated disagreements. Only very limited pooling of data was possible. The primary outcomes were function (for example, independence in mobility and activities of daily living) and adverse events, including mortality.

**MAIN RESULTS:**

We screened 1290 records and found only three trials involving 154 female participants, all of whom were aged above 65 years and had had hip fracture surgery. All studies had methodological shortcomings that placed them at high or unclear risk of bias. Because of this high risk of bias, imprecise results and likelihood of publication bias, we judged the quality of the evidence for all primary outcomes to be very low.

These trials tested two comparisons. One trial had three groups and contributed data to both comparisons. None of the trials reported on patient acceptability of the intervention.

Two very different trials compared anabolic steroid versus control (no anabolic steroid or placebo). One trial compared anabolic steroid injections (given weekly until discharge from hospital or four weeks, whichever came first) versus placebo injections in 29 "frail elderly females". This found very low quality evidence of little difference between the two groups in the numbers discharged to a higher level of care or dead (one person in the control group died) (8/15 versus 10/14; risk ratio (RR) 0.75, 95% confidence interval (CI) 0.42 to 1.33; P = 0.32), time to independent mobilization or individual adverse events. The second trial compared anabolic steroid injections (every three weeks for six months) and daily protein supplementation versus daily protein supplementation alone in 40 "lean elderly women" who were followed up for one year after surgery. This trial provided very low quality evidence that anabolic steroid may result in less dependency, assessed in terms of being either dependent in at least two functions or dead (one person in the control group died) at six and 12 months, but the result was also compatible with no difference or an increase in dependency (dependent in at least two levels of function or dead at 12 months: 1/17 versus 5/19; RR 0.22, 95% CI 0.03 to 1.73; P = 0.15). The trial found no evidence of between-group differences in individual adverse events.

Two trials compared anabolic steroids combined with another nutritional intervention ('steroid plus') versus control (no 'steroid plus'). One trial compared anabolic steroid injections every three weeks for 12 months in combination with daily supplement of vitamin D and calcium versus calcium only in 63 women who were living independently at home. The other trial compared anabolic steroid injections every three weeks for six months and daily protein supplementation versus control in 40 "lean elderly women". Both trials found some evidence of better function in the steroid plus group. One trial reported greater independence, higher Harris hip scores and gait speeds in the steroid plus group at 12 months. The second trial found fewer participants in the anabolic steroid group were either dependent in at least two functions, including bathing, or dead at six and 12 months (one person in the control group died) (1/17 versus 7/18; RR 0.15, 95% CI 0.02 to 1.10; P = 0.06). Pooled mortality data (2/51 versus 3/51) from the two trials showed no evidence of a difference between the two groups at one year. Similarly, there was no evidence of between-group differences in individual adverse events. Three participants in the steroid group of one trial reported side effects of hoarseness and increased facial hair. The other trial reported better quality of life in the steroid plus group.

**AUTHORS' CONCLUSIONS:**

The available evidence is insufficient to draw conclusions on the effects, primarily in terms of functional outcome and adverse events, of anabolic steroids, either separately or in combination with nutritional supplements, after surgical treatment of hip fracture in older people. Given that the available data points to the potential for more promising outcomes with a combined anabolic steroid and nutritional supplement intervention, we suggest that future research should focus on evaluating this combination.

The full-text of this review (English), the abstract (English and French) and a plain language summary (for patients and consumers, in English and French) are available from: http://onlinelibrary.wiley.com/doi/10.1002/14651858.CD008887.pub2/full
